# Detection of *Salmonella* in Food Matrices, from Conventional Methods to Recent Aptamer-Sensing Technologies

**DOI:** 10.3390/foods8090371

**Published:** 2019-09-01

**Authors:** Nathalie Paniel, Thierry Noguer

**Affiliations:** 1 Laboratoire BAE, Université de Perpignan Via Domitia, 52 Avenue Paul Alduy, 66860 Perpignan, France; 2 Unité EMaiRIT’S, Centre Technique de la Conservation des Produits Agricoles (CTCPA), Site Agroparc, 449 Avenue Clément Ader, BP21203, 84911 Avignon, France; 3 Laboratoire de Biodiversité et Biotechnologies Microbiennes, USR 3579, Sorbonne Universités (UPMC) Paris 6 et CNRS, Observatoire Océanologique, 66650 Banyuls-sur-Mer, France

**Keywords:** *Salmonella*, food matrices, aptamers, biosensors, standard methods

## Abstract

Rapid detection of the foodborne pathogen *Salmonella* in food processing is of crucial importance to prevent food outbreaks and to ensure consumer safety. Detection and quantification of *Salmonella* species in food samples is routinely performed using conventional culture-based techniques, which are labor intensive, involve well-trained personnel, and are unsuitable for on-site and high-throughput analysis. To overcome these drawbacks, many research teams have developed alternative methods like biosensors, and more particularly aptasensors, were a nucleic acid is used as biorecognition element. The increasing interest in these devices is related to their high specificity, convenience, and relative rapid response. This review aims to present the advances made in these last years in the development of biosensors for the detection and the quantification of *Salmonella*, highlighting applications on meat from the chicken food chain.

## 1. Introduction

### 1.1. Salmonella and Food Contamination

Food contamination by pathogenic bacteria is a significant public health concern for consumers worldwide. The economic consequences are also significant for the producers and the industry [1,2]. In the European Union, the second most frequently bacterial genus involved in gastrointestinal outbreaks in human is *Salmonella* and more particularly the species *Salmonella* Enteritidis (*S*. Enteritidis) and *Salmonella* Typhimurium (*S*. Typhimurium) [2,3,4,5,6,7,8,9]. In France, almost half of the the 1500 collective foodborne infections recorded each year, are caused by bacteria belonging to the genus *Salmonella*. Among the different serotypes, *S*. Enteritidis and *S*. Typhimurium particularly predominate in poultry meat foods [10,11,12,13,14,15,16]. These bacteria, which are non-typhoid *Salmonella* are responsible for salmonellosis, an infectious disease-causing acute gastroenteritis [17,18,19,20], which occurs in 95% of cases through consumption of contaminated food, especially meat and eggs. Non-typhoid human salmonellosis is considered to be a zoonotic disease, the main reservoir of *Salmonella* being the gastrointestinal tract of mammals (cattle and pigs) and birds [21]. Livestock carrying these bacteria rarely develop symptoms, making them almost impossible to detect. Since these bacteria are able to survive out of their natural habitat, some fresh products like fruits and vegetables can be contaminated by the feces of infected animals. Preventive approaches such as hazard analysis and critical control point (HACCP) can considerably reduce the survival of pathogens during the process of food handling, preparation and storage. Therefore, identification and detection of microorganisms in the food processing play an important role for preventing food outbreaks.

### 1.2. Salmonella Detection and Quantification by Conventional Methods

**Figure 1:**
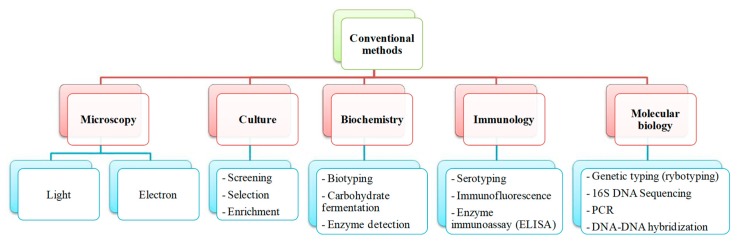
Conventional methods used for food borne pathogenic bacteria detection.

**Figure 2:**
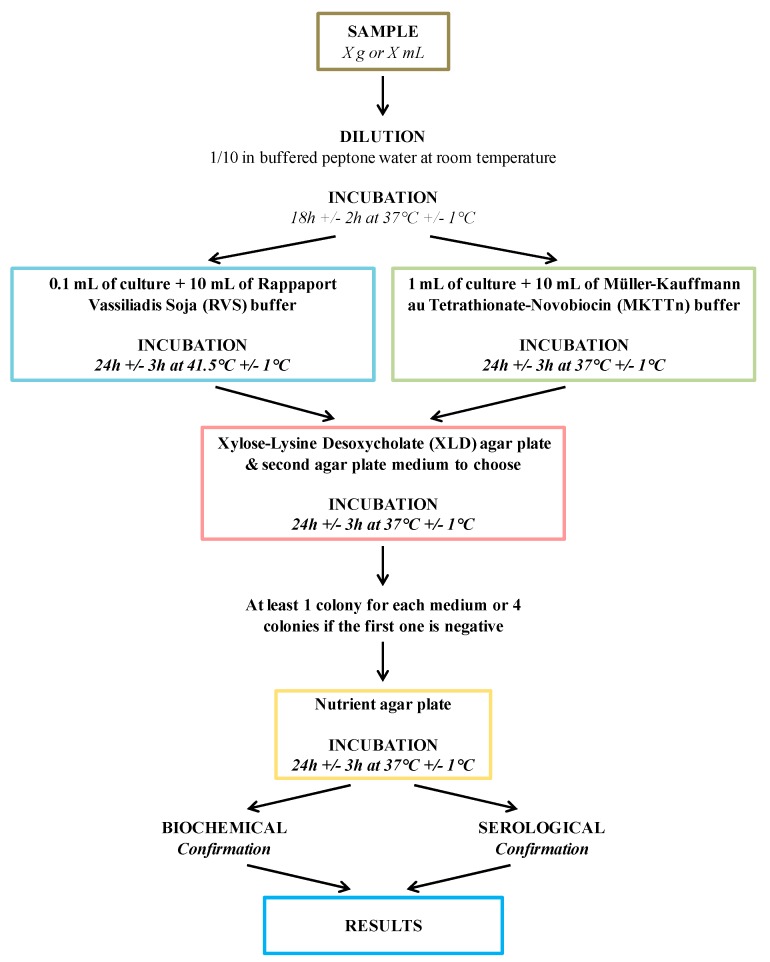
International standard NF EN ISO 6579. This international standard is a horizontal method used for the detection of *Salmonella*, including *S*. Typhi and *S*. Paratyphi, in products intended for human consumption or animal feed and in environmental samples in the area of production and handling of food.

Conventional methods for isolation of bacteria are based on cultures grown on differential agar media and subsequent colony counting (Figure 1) [22,23,24,25]. The basic steps for the detection of *Salmonella* in food include a pre-enrichment in buffered peptone water and an enrichment in selective media, followed by isolation on differential media and serological confirmation [26] (NF/EN/ISO 6579) (Figure 2). However, interpretation of these tests is often difficult, making the method laborious and time consuming. Moreover, two to four days are required to obtain the initial results, and four to six additional days are necessary to confirm a positive result [1,2,8,19,22,27,28]. During this period of time, commercialization of these food stocks is forbidden.

Other drawbacks of conventional cultural methods are related to their low sensitivity, risk of microbial contamination resulting in the growth inhibition of bacteria of interest, and presence of viable but non-culturable bacteria (VBNC). The consequences of the presence of VBNC include underestimation of viable cells number or an impossibility to isolate the pathogens from the sample [8,29,30,31,32]. A VBNC state is commonly found in environmental and food samples due to starvation of bacteria and to a large variety of stressful conditions, including growth inhibiting temperature, anoxia, and non-optimal pH and salinity [33]. In food, it was reported that a VBNC state was, in some cases, directly induced by food disinfection techniques [34,35,36]. Because these bacteria cannot be detected by common techniques, they present an increased risk for consumers [37]. Some authors have already shown that VBNC cells of *Salmonella* Typhi (*S*. Typhi), but also *Escherichia coli* (*E. coli*) and *Legionella pneumophila (L. pneumophila)*, as well as other bacteria, are still able to produce virulence factors [38,39,40]. Therefore, in order to reach more robust results, standard microbiological count methods are often combined with other automated or semiautomated detection techniques involving DNA, antibody, or biochemical approaches (Figure 1). However, several drawbacks are still encountered with these traditional methods (Table 1), and there is still a need for developing more rapid, sensitive, and specific techniques for the detection and quantification of pathogens.

The development of new techniques with faster response time, better sensitivity, and selectivity is very important to ensure consumers safety. Immunological methods based on specific antigen and antibody binding have been developed for the detection of *Salmonella* [41,42,43]. For bacteria, the targets of immunological assays (IAs) are either whole bacterial cells or specific cellular components like lipopolysachharides or other biomolecules of bacterial outer membrane [1]. Among them, enzyme immunoassay (EIA) [44], enzyme linked immunosorbent assay (ELISA) [45], flow injection immunoassay [46], immunochromatography strip test (ICG) [47], and immunomagnetic separation [20,48] have been extensively used. The most common format used for pathogens detection is the ELISA, in sandwich format with direct or indirect labeling. Generally, the limit of detection (LOD) of the ELISAs developed for pathogens ranges from 10^4^ to 10^5^ CFU/mL, with an analytical time of 48 h, due to the need, for food samples, of a pre-enrichment step [49,50]. Magliulo et al. [42] have developed a multiplex sandwich chemiluminescent enzyme immunoassay for the simultaneous detection of *E. coli* O157:H7, *Yersinia enterocolitica (Y. enterocolitica)*, *S*. Typhimurium, and *Listeria monocytogenes (L. monocytogenes)*. A new 96-well polystyrene microtiter plate was used in which each main well contains four subwells, where monoclonal antibodies, specific for each bacteria, were grafted. When introducing samples containing the targeted bacteria into the modified wells, bacteria capable of specifically binding to the corresponding monoclonal antibody present in one of the four secondary wells were trapped. Then, a mixture of peroxidase-labeled polyclonal antibodies was allowed to bind to the bound bacteria and the peroxidase activity was measured after addition of an enhanced luminol-based chemiluminescent cocktail using a low-light charge-coupled imaging device. The limit of quantification (LOQ) was in the order of 10^4^ to 10^5^ CFU/mL for each species [42]. Generally, immunological methods permit real-time detection of microorganisms, within shorter times as compared to cultural methods. However, these methods have some disadvantages including low affinity, poor sensitivity, and potential interferences from contaminants [51] (Table 1). 

Polymerase chain reaction (PCR) based methods have also been applied for the detection and identification of bacteria in a large variety of samples [52,53,54,55,56,57,58,59,60,61]. Compared to other conventional methods, PCR-based methods have shown better specificity, higher sensitivity, shorter analysis time, and better accuracy [29] (Table 1). *Salmonella* have been detected using classical PCR, real-time PCR, multiplex PCR, and reverse transcriptase PCR (RT-PCR) [62,63,64,65,66,67,68,69,70,71], sometimes in association with other techniques like immunomagnetic separation [49,67,72]. All these methods can be applied to in situ, real-time monitoring for many applications, including detection and characterization of *Salmonella* in poultry, poultry products, and feeds. These techniques enable the detection of subdominant bacterial populations, even in the absence of selective enrichment medium and in the presence of other dominant populations. However, an enrichment step of a few hours is sometimes necessary before performing qPCR to fulfil the requirements of national and international legislations for foodstuffs [73]. A study of the specificity of the PCR detection method under varied enrichment protocols confirmed this fact [2]. During this study, chicken meat samples (ground, boneless/skinless breast meat, and bone-in breast meat with skin) from retail groceries were pre-enriched in buffered peptone water. A couple of primers, ST11 and ST15, designed by Aabo et al. [74] were used to amplify a region of the random fragment (429 bp) specific to all *Salmonella* spp. The use of buffered peptone water for pre-enrichment, and Rappaport-Vassiliadis and tetrathionate Hajna broths for selective enrichment allowed a specificity of 100% to be obtained. The use of only one pre-enrichment with buffered peptone water decreased the sensitivity to 85%, while no pre-enrichment resulted in an impossibility to detect positive samples. The same authors have demonstrated that a minimum pre-enrichment step of 12 h was necessary to detect *Salmonella* by PCR at a limit of 100 CFU/mL [2]. Oliveira et al. [69] developed a PCR for the generic detection of *Salmonella* spp. and the identification of *S*. Enteritidis, *S*. Gallinarum, *S*. Pullorum, and *S*. Typhimurium in samples collected in poultry field. For each sample, a selective enrichment was performed in Rappaport-Vassiliadis broth, followed by DNA extraction and PCR amplification. The LOD obtained by PCR for *Salmonella* at the genus level was two cells for *S*. Typhimurium, eight cells for *S*. Enteritidis, 1.1 × 10^3^ cells for *S*. Gallinarum, and 1.8 × 10^5^ cells for *S*. Pullorum. At the serovar level, the LOD was seven cells for *S*. Typhimurium, 1.2 × 10^3^ cells for *S*. Enteritidis, 4.4 × 10^7^ cells for *S*. Gallinarum, and 1.8 × 10^6^ cells for *S*. Pullorum. These results were obtained in 48 h instead of seven days. Similarly, Makino et al. [75] developed a PCR detection system for the specific detection of twenty serovars of *Salmonella* by targeting the *Salmonella* enterotoxin gene (*stn*). The PCR was realized after an enrichment step with trypticase soy broth or *Salmonella* enrichment broth. A detection limit of one cell per one gram of fecal and minced-meat samples was obtained.

To increase the accuracy and to decrease the time of analysis, some multiplex PCR methods were developed allowing the simultaneous identification of multiple pathogens in one sample within a single reaction [59,64,65,76,77,78,79]. Sharma and Carlson [80] developed a multiplex fluorogenic PCR assay for the simultaneous detection of *Salmonella* and *E. coli* O157:H7 in meat and feces. In the case of *Salmonella*, the set of primer was designed to amplify a junctional segment of virulence genes *sipB* and *sipC*. The LOD was lower than 10 CFU/g in meat or feces artificially inoculated with *Salmonella* and grown during six to 18 h in an enrichment broth. In a similar way, Yu et al. [79] developed a multiplex-PCR (m-PCR) for the simultaneous detection of *Salmonella* spp., *S. aureus*, and *L. monocytogenes* using as target genes, *xcd*, *vicK* and LMOf 2365-2721, respectively. A detection limit of 10^3^ CFU/mL was achieved for the simultaneous detection of the three pathogens. The m-PCR method has been used to detect and identify *Salmonella* in poultry samples. For example, Soumet et al. [59] developed a multiplex PCR-based assay (m-PCR) with the following three sets of primers: ST11-ST15 were selected for the specific detection of the genus *Salmonella* (Aabo et al. [74])*;* S1-S4 [59] were specific for *S*. Enteritidis from a gene associated with virulence [81]; while Fli15-Typ04 primers [59] were chosen from the *fliC* gene, specific for *S*. Typhimurium. As described for classical PCR, the samples from swabs of poultry houses were pre-enriched in phosphate-buffered peptone water for 24 h prior to multiplex PCR assay. These authors showed that a poor sensitivity (10^7^ *Salmonella*/mL) or even no amplified product was obtained if m-PCR was applied directly from a pre-enrichment broth. An additional culture on a modified semi-solid Rappaport-Vassiliadis (MSRV) medium was thus performed to obtain similar results to those obtained from bacteriological methods [59]. The resulting MSRV-PCR assay provided a result on *Salmonella* within 48 h. The authors estimated that the low sensitivity of direct m-PCR assay may be explained by the presence of fewer *Salmonella* in pre-enrichment broth, which was lower than the LOD evaluated as 10^4^ *Salmonella*/mL).

Recently, Xiong et al. [82] developed a one-step multiplex PCR assay for *Salmonella* to simultaneously identify and discriminate Pullorum and Gallinarum biovars. The genes targeted by this m-PCR were the genes *stn, I137_0860* and *ratA*. The unique gene *I137_08605*, present only in biovars Gallinarum and Pullorum, was a common feature shared by these biovars, but was not present in any other known *Salmonella* serovars or species. A deletion within the biovar Pullorum was evidenced by the sequence analysis of *ratA ROD* in serovar Gallinarum strains. A total of 124 strains of various *Salmonella* serovars and 42 strains of different non-*Salmonella* pathogens were tested, and the results showed that *S*. Pullorum and *S*. Gallinarum could be identified and discriminated accurately. Moreover, this m-PCR assay had a specificity of 100% and was able to quantify as low as 67.4 pg/mL of genomic DNA and detect 100 CFU. Heymans et al. [83] developed a multiplex quantitative PCR for the simultaneous detection and the differentiation of *Salmonella* species, *S*. Typhimurium, and *S*. Enteritidis in various food matrices, in which these bacteria were detected by targeting the *invA* gene, the *STM4200* gene, and the *SEN1392* gene, respectively, for which three sets of primer and probe were designed. Inclusivity and exclusivity of 225 *Salmonella* and 34 non-*Salmonella* isolates were evaluated. The inclusivity of the multiplex qPCR was 100% for all *Salmonella* isolates, including 72 and 53 isolates from *S*. Typhimurium and *S*. Enteritidis, respectively. The exclusivity for *Salmonella* spp., *S*. Typhimurium, and *S*. Enteritidis was 100%, 94.6%, and 100%, respectively. Non-*Salmonella* isolates led to negative results. The LOD was determined for various matrices including poultry, meat, egg, herbs, and powdered milk. The LOD values for qPCR and conventional culture methods (ISO and MSRV) were in the same order, allowing the detection of *Salmonella* at approximately 10 CFU/25 g.

Quantitative (real-time) PCR (qPCR) has often been reported for the quantification of *Salmonella* in poultry samples [84,85,86,87]. The development of fluorescence-based techniques involving molecular beacons, TaqMan, and SYBR Green probes has allowed increasing the sensitivity of these assays. Malorny et al. [87] developed a duplex 5’ nuclease TaqMan qPCR assay for the specific detection of *S*. Enteritidis in chicken carcass rinses and eggs. The authors have designed specific primers and a TaqMan probe to target the *Prot6e* gene located on the *S*. Enteritidis specific 60 kb virulence plasmid. They also used a second primer TaqMan probe set for the simultaneous detection of the *invA* gene. The detection limit was less than 3 CFU/50 mL of carcass rinse or 10 mL of eggs. The sensitivity and specificity as compared to the traditional culture-based detection method and serotyping were both 100% [87]. Similarly, Hein et al. [88] developed a qPCR TaqMan assay allowing the detection and the quantification of *Salmonella* in different artificially contaminated foods after 16 h of enrichment in buffered peptone water or universal pre-enrichment broth. The method was able to detect 5 CFU in 25 g of chicken meat, 2.5 CFU in 25 g of salmon and minced meat, and 5 CFU in 25 mL of raw milk [88]. Ellinggson et al. [89] developed a rapid real-time quantitative PCR for the detection of *Salmonella* spp. in ready-to-eat beef products. The primers were designed to amplify a 251-base pair product from the junction to *SipB* and *SipC*. One of the two probes used for the hybridization was labeled with fluorescein. This method allowed the detection of one colony of *Salmonella* in 1 mL of food product within 12 h. A control was realized with visual immunoprecipitate and cultural methods and a correlation of 100% was obtained between these methods and the developed molecular method [89]. Cremonesi et al. [90] developed an individual TapMan^®^ real-time PCR for the simultaneous detection of 20 foodborne pathogens including *Listeria* spp., *Salmonella* spp., *Shigella* spp., *Escherichia coli*, *Campylobacter* spp., *Clostridium* spp., and *Staphylococcus aureus* in complex alimentary matrices such as milk, cheese, and meat. The accuracy of detection was determined by using ATCC strains as positive and negative controls. For each assay, the achieved sensitivity was of 1pg of genomic DNA, which was equivalent to approximately one CFU. The working ranges of this assay was between 10^8^ CFU/g to 10^4^ CFU/g for *S. enterica* and the other studied strains. Four hours were required to perform the test. Recently, Bai et al. [91] developed a multiplex real-time PCR for the detection and the quantification of *Salmonella enterica* from cattle lymph nodes. The most conserved molecular targets of *S. enterica* retained for the development of the assay were the genes *invA* and *pagC*. Potential false negative responses were eliminated by adding as an internal control the 18S rRNA gene using a lymph node spiked with 10-fold dilutions of a *S*. Typhimurium culture. To carry out the selection of primers and probes, the authors used the DNA sequences available for *invA, pagC*, and 18S rRNA genes, as well as three *Salmonella* serotypes (*S*. Typhimurium, *S*. Anatum, and *S*. Montevideo). For each target and for all three serotypes the correlation coefficient of the standard curves was higher than 99% and the efficiency of the qPCR amplification was comprised between 93% and 110%. An evaluation of the specificity of the assay was carried out using cultural method versus qPCR on 36 *Salmonella* strains representing 33 serotypes, 38 *Salmonella* strains of unknown serotypes, 252 *E. coli* strains representing 40 serogroups, and 31 other bacterial strains representing 18 different species. A collection of 647 cattle lymph node samples from steers were tested and compared to the culture method of detection. The qPCR analysis of pre-enriched and enriched lymph nodes showed a *Salmonella* prevalence of 19.8% and 94.9%, respectively. A majority of qPCR positive pre-enriched samples were at concentrations between 10^4^ and 10^5^ CFU/mL. Culture method allowed detecting *Salmonella* in 7.7% and 80.7% of pre- and post-enriched samples, respectively, while 96.0% of pre-enriched and 99.4% of post-enriched culture-positive samples were also positive by qPCR.

During the 2000s, a new molecular method called viability PCR (v-PCR) was developed for the detection of viable cells [92,93,94,95]. This technique couples together PCR or qPCR with the use of intercalating dyes such as propidium monoazide (PMA), ethidium monoazide (EMA), or a mix of photo-reactive azide forms of phenanthridium (PEMAX). The v-PCR method is based on the integrity of the bacterial cells, PMA, EMA, and PEMAX that are viability dyes named also intercalating dyes which penetrate only into compromised membrane cells. Once inside the cell, these dyes can be covalently linked to DNA by photoactivation. In this case, the irreversible binding of photoactivated EMA or PMA to DNA inhibits the amplification of the DNA from dead bacteria [92,93]. However, as for conventional PCR-based DNA amplification methods, the efficiency of the v-PCR may be affected by different events of the detection process, such as a decrease of dye efficiency due to chemical adsorption, ineffective photoactivation due to the presence of organic compounds, degradation of nucleic acids, or inhibition of polymerase activity. The concentration of cells, turbidity, salt concentration, and pH can also interfere with the v-PCR results [92,96,97]. Indeed, Martin et al. [98] highlighted an inhibition effect dependent on the PCR amplification product length. Three PCR targets of 95, 285, and 417 bp combined with a PMA pretreatment to enumerate viable *Salmonella* cells in cooked ham were studied and only the longer product achieved suppression of 10^8^ CFU/g of heat-killed cells. A major limitation of the of the v-PCR method for a wider use and its application in routine quality control is the incomplete exclusion of dead micro-organisms leading to false positive signals, in particular with high background of dead cells [95,98]. To improve the efficiency of the v-PCR, Dinh Thanh et al. [95] combined the PEMAX dye with a double tube change and a double photo-activation step. These approaches allow the neutralization of DNA signals of up to 5.0 × 10^7^ dead cells per sample from both pure culture and artificially contaminated food samples. There results show the potential of vPCR for high throughput detection of live *Salmonella* cells in food samples, minimizing false positive signals [95].

As for classical PCR and m-PCR methods, an additional immunoprecipitation step may be necessary to overcome the problems of inhibition induced by the food matrix. For instance, Lynch et al. [48] studied the ability of an automated immunomagnetic separation system using anti-*Salmonella*-modified Dynabeads to detect *Salmonella* spp. in poultry environmental samples as compared with a standard culture-based method. The automated immunomagnetic separation system was more reliable for the detection of *Salmonella* in artificially inoculated enrichment broth at a low level and the sensitivity was 15.5% higher than the cultural method [48]. Similarly, Josefsen et al. [99] developed a 12 h real-time PCR assay for *Salmonella* in meat and poultry, based on an 8 h pre-enrichment followed by an automated DNA extraction with the help of paramagnetic particles. A validation of the established method was realized with 100 minced meat and poultry samples and with artificially inoculated reference samples and the results showed a relative accuracy of 99%, a relative sensitivity of 98%, and a relative specificity of 100%. [99]. Recently, Taha et al. [100] compared immunomagnetic separation (IMS) followed by culture in CHROMagar Plus media, ELISA, and real-time PCR methods for the detection and quantification of *S*. Typhimurium ATCC 13311 in chicken wing samples (25 g) spiked with six different concentrations of bacteria ranging from 10^6^ to 10^1^. Spiked samples were incubated in buffered peptone water for 4 h and the different methods were applied. For the RT-PCR, the primers target was the *invA* gene. In comparison with the usual four-day cultivation method, the culture on CHROMagar medium post IMS showed, in 23 h, the presence of light to purple colonies corresponding to a *Salmonella* concentration of 1.6 CFU/mL with high sensitivity (99%). The combination of the IMS with the ELISA method also demonstrated a high sensitivity (75%) allowing a *Salmonella* concentration of 1.6 × 10^3^ CFU/mL to be counted in 8 h while minimizing cross-reactivity, particularly with many *Enterobacteraceae*. A higher sensitivity and a faster resolution time (7 h) were obtained by combining the IMS with RT-PCR to detect a *Salmonella* concentration of 1.6 CFU/mL. Therefore, the sensitivity of the IMS-RT-PCR and IMS-CHROMagar was higher than that of the IMS-ELISA [100].

The possible limitation of the PCR-based techniques lies in the difficulty of distinguishing between viable and nonviable cells, as they both contain DNA. Moreover, PCR-based methods often lead to false positives or false negative results [101], induced in some cases by the inhibition of amplification reaction by matrix compounds (lipids and proteins in meat and dairy, polysaccharides and polyphenols in vegetable and fruits), or by the degradation of the target nucleic acid sequences in the sample [92,102,103,104,105]. Cross-contamination between samples may also occur. Another major problem is the presence of PCR inhibitors in food samples [59]. Such inhibition can also be due to the nature of the enrichment broth [106]. Therefore, some adjustments can be made including the use of clean-up methods and the addition of facilitators of the PCR reaction [92,96,104,107,108]. Nowadays, internal amplification controls to identify PCR inhibition are routinely used to confirm the efficacy of the sample preparation and the success of the clean-up steps [105,109]. To overcome this problem, removal of inhibitors from DNA extract or separation of bacteria from the samples seems to be the solutions. Many methods have been reported for sample preparation, including filtration, centrifugation, enzyme treatment, sample dilution, use of detergents and organic solvents, and immunomagnetic separation [103].

The team of Resendiz-Nava [110] observed during a surveillance program for *Salmonella* that conventional *invA* PCR assay led false positive signals for some bacterial isolates. It was thus decided to perform an evaluation of the performance of the other published primers targeting the *invA* gene. In this aim, a collection of strains of *Citrobacter* spp., *E. coli*, and *Serratia* spp. recovered from poultry meat was tested in PCR targeting the *invA* gene, but all the selected *invA* gene primers generated nonspecific signals. Comparable results have been reported by other teams like Malorny et al. [109] and Scholz et al. [111] in reactions containing genomic DNA from non-*Salmonella* isolates. Recent studies have reported a high specificity for *invA* PCR assays [83,91] but, unlike the study of Resendiz-Nava et al. [110], the experiments were carried out using DNA obtained from type strain collections. Resendiz-Nava et al. [110] and Kloska et al. [112] studies also revealed that, due to its high specificity and amplicon size (~90 bp), the primer set ttr-6 + ttr-4 targeting the *ttrA*/C genes (tetrathionate reductase subunit A/C) allowed discriminating between *S. enterica* and non-*Salmonella* isolates. Comparable results were reported by Malorny et al. [113] using a set of 110 *S*. *enterica* strains, representing 38 different serovars and 87 non-*Salmonella* strains. Therefore, PCR assays based on *invA* gene amplification were not reliable for *Salmonella* detection. False positive results were commonly obtained from *Citrobacter* spp., *E. coli*, and *Serratia* spp. isolates. Other loci, such as *ttrA/C* genes, should be, thus, used for the accurate and reliable detection of this pathogen.

Immunomagnetic separation (IMS) is based on super-paramagnetic particles coated with antibodies specific to the targeted bacteria. These modified paramagnetic particles can be introduced in a culture medium or food sample to allow the capture and the concentration of the bacteria. In a similar manner, magnetic particles modified with bacteriophages have been recently used for the preconcentration of *Salmonella* from milk samples [114]. In this work, the detection was realized using specific anti-*Salmonella* antibodies conjugated to horseradish peroxidase as an optical reporter. A detection limit of 19 CFU/mL of milk samples was achieved within 2.5 h without any pre-enrichment [114]. IMS is widely used due to several advantages like preconcentration of the target bacteria into small volumes, reduction of matrix effect due to food components, and the simplification of the pre-enrichment step. However, the assay efficiency is highly dependent on the antibody’s affinity and specificity against the targeted bacteria. A high cross-reactivity of antibody may increase the risk of false positive results [1,49,115].

Next generation sequencing (NGS) or whole genome sequencing (WGS) is transforming the laboratory practices for foodborne disease investigations, and more particularly *Salmonella* contaminations. Due to their lack of characterization of the *Salmonella* strains, and the difficulty of tracking and delimiting the source of contamination, the use of conventional methods have been gradually replaced by WGS [104]. Vohra et al. [116] developed a new approach based on WGS to replace traditional methods such as colony subculture and serogroup identification. The method was based on the use of the inherent differences in the genomes of *S. enterica* serovars and quantified the dynamics of mixed serovar infections in vivo and their survival within the bovine lymphatic system to predict their zoonotic potential. The strength of this approach is that the study of the bacterial strains does not involve genetic manipulation and significantly reduces the number of animals required for in vivo study of mixed infections.

**Figure 3:**
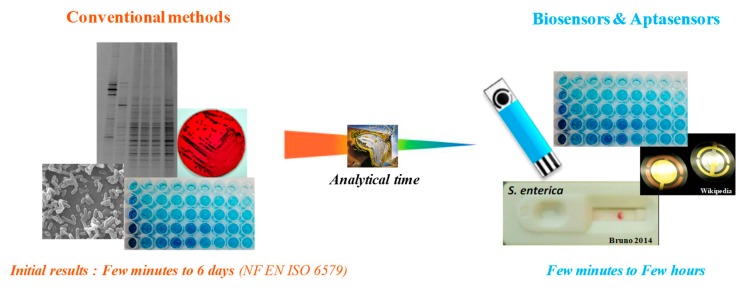
Comparison of the analytical time of the conventional methods versus the biosensors and the aptasensors for the detection of foodborne bacteria.

During the last twenty years, biosensors have emerged as promising alternative tools for environmental monitoring, clinical diagnostic and food analysis. They are based on the tight association of a sensitive biological element and a physical interface, called transducer, which allows obtaining an output signal. Biosensors are easy to use, versatile, low cost, portable and allow a real-time detection (Figure 3). Moreover, they can be used in dirty environments with minimal sample preparation [8,27,117,118,119,120,121,122,123,124,125,126,127,128,129,130,131,132,133]. Basically, biosensors for bacterial detection generally use antibodies as recognition element, but more and more devices are now developed based on nucleic acids, and more specifically DNA aptamers.

In the first part, this review explores and summarizes the immunosensors described for *Salmonella* detection and quantification in food matrices and, if possible, in food from chicken chain. In a second part, aptamers-based methods for *Salmonella* detection in the same matrix are described.

## 2. Biosensors for *Salmonella* Detection and Quantification

**Figure 4:**
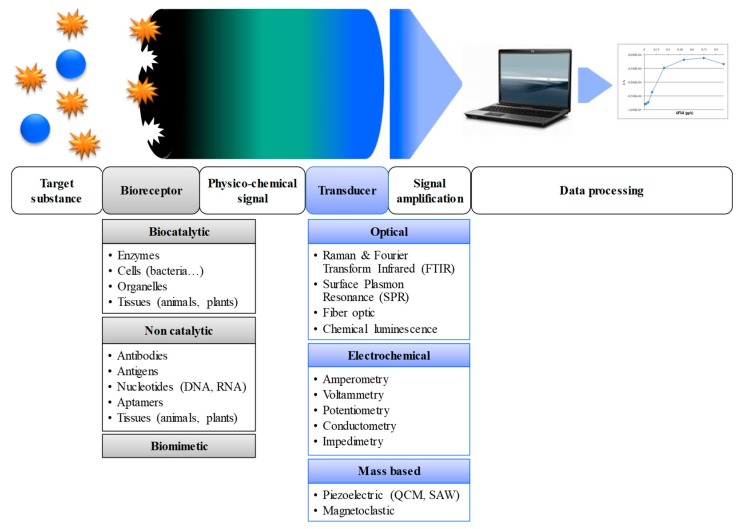
Synoptic representation and classification of biosensors.

A biosensor is an analytical tool consisting of the close association of two elements (Figure 4). The biological recognition element, sometimes called bioreceptor, is capable of interacting specifically with the target molecule, while the transducer allows converting the biological signal into measurable signal. Bioreceptors may be biocatalytic when they allow the transformation of target molecule (enzymes, whole cells, organelles, etc.), or they may be non-catalytic when affinity antigens, antibody, nucleic probes, aptamer, or tissue are used to simply bind the target molecule. The bioreceptor is generally immobilized in close contact to the transducer surface and it must have a high specificity and sensitivity towards his target to allow a response in a short time. According to the method of transduction, biosensors may be divided in the following three main categories: optical, electrochemical, and mass-sensitive sensors. Among the various reported biosensors, immunosensors have been the most used for the detection of *Salmonella*.

Biosensors for bacteria detection [25,134] must allow the detection of a single bacterium in a reasonably small sample volume (1–100 mL), and they should be able to discriminate between individual bacterial species and other microorganisms or cells, and even other strains of the same species. The precision may be less than 10%, with an assay time of between five to 10 min for a single test. Ideally, a microbial sensor should be able to discriminate between live and dead cells and should operate without pre-enrichment. However, biosensors described to date in the literature could not meet all these requirements.

### 2.1. Optical Biosensors

Optical biosensors are based on the measurement of a light signal (visible, ultraviolet and infrared) resulting from a chemical and biological reaction, which is captured by an appropriate transducer and converted into data format. Optical biosensors are represented by surface plasmon resonance-based sensors (SPR), colorimetry-, fluorometry-, bioluminescence-, photoluminescence-, and chemiluminescence-based sensors.

#### 2.1.1. Surface Plasmon Resonance Biosensors

Surface plasmon resonance (SPR) is an optical technique for detecting the interaction of two different molecules in which one is mobile and the other is fixed on a thin gold film. SPR spectroscopy is a mass-sensitive sensor that detects the mass change in association with the change in the refractive index at the surface due to the molecular binding event. Because the method strictly detects mass, there is no need to label the interacting components, thus eliminating possible changes of their molecular properties. Lan et al. [135] developed an optical surface plasmon resonance (SPR) biosensor to detect the presence of *S*. Typhimurium in chicken carcass. Their SPR-biosensor allows the detection of *S*. Typhimurium at 1 × 10^6^ CFU/mL in chicken carcass (Table 2). Other teams have reported the detection of *S*. Typhimurium in a similar range of 1 × 10^5^ to 1 × 10^7^ CFU/mL also using a SPR biosensor [136,137] (Table 2).

#### 2.1.2. Fluorescence-Based Sensors

Fluorescence resonance energy transfer (FRET) allows the measurement of the distance between two chromophores (donor-acceptor pair). The transfer process is effective only if the distance between the donor and the acceptor pair is smaller than 10 nanometers.

The detection of *S*. Typhimurium was achieved by a FRET optical fiber tip sensor using a *Salmonella* antibody labeled with a FRET-donor fluorophor, Alexa Flur 546, and a protein G labeled with the FRET-acceptor fluorophore Alexa Fluor 594 [138]. The binding of *S*. Typhimurium induced conformation changes of the antibody, resulting in a decrease of distance between donor and acceptor and an increase of fluorescence. The LOD of this FRET sensor was 10^3^ cells/mL. The fiber probes were applied for detecting *S*. Typhimurium at 10^5^ CFU/g in homogenized pork samples [138] (Table 2). More recently a team compared a fiber optic immunosensor and a light scattering sensor “BARDOT” (bacterial rapid detection using optical scattering technology) for detecting *S*. Enteritidis and *S*. Typhimurium in naturally contaminated poultry [139]. Using the fiber optic sensor, a detection limit of 10^3^ CFU/mL was obtained in less than 12 h for *S. enterica*, after selective enrichment in Rappaport-Vassiliadis broth. The enriched samples were plated onto selective XLT4 agar and after 13–15 h of incubation the colonies were scanned using BARDOT. Each individual colony scatter pattern was compared to a previously designed scatter image library, *S. enterica* was identified, and the results were obtained within 24 h. The authors [139] validated the used of BARDOT to detect *S. enterica* serovars (Table 2). Song et al. also developed a biosensor for the detection of *S*. Enteritidis by FRET using a nicking enzyme and carbon nanoparticles (CNPs) [142]. The surface of the CNPs was modified by the grafting of a particular ssDNA containing two consecutive sequences. The first sequence was complementary to 16S rRNA and the second was complementary to a molecular beacon tagged with a black hole quencher (BHQ1), which was recognized by the nicking endonuclease. When the DNA of the *S*. Enteritidis was added to the CNTs grafted with ssDNA, the connection between the CNT and the ssDNA was broken by forming a dsDNA between the *S*. Enteritidis 16S rRNA sequence and its complementary DNA. Therefore, a dsDNA was obtained which was connected to the ssDNA complementary sequence of the molecular beacon tagged with a BHQ1. The addition of the nicking enzyme induced the cleaving of the molecular beacon from the DNA, and a fluorescence signal appeared. In the absence of target, the molecular beacon is not hybridized and cleaved because the CNTs and the particular ssDNA sequences are linked with a covalent bond, which prevents the release of the sequence from the CNTs. This biosensor had a linear response ranging from 10^2^ to 3 × 10^3^ CFU/mL in water and from 1.5 × 10^2^ to 3 × 10^3^ CFU/mL in milk (Table 2). Another team [143], developed an evanescent wave-based fiber optic immunosensor for the simultaneous detection of *S*. Enteritidis, *L. monocytogenes*, and *E. coli* O157:H7 in meat (beef, chicken, and turkey). A sandwich format was used where biotinylated polyclonal antibodies were grafted on the optical waveguides and were exposed to the bacterial suspensions or enriched food. After 2 h of contact, Alexa Fluor 647-labeled monoclonal antibodies were added and the fluorescence was quantified. This biosensor was able to detect each pathogen, individually or in mixtures, with a LOD near 10^3^ CFU/mL (Table 2). Kim et al. [140] developed a microfluidic nanobiosensor for the detection of *S*. Typhimurium based on the use of quantum dot nanoparticles. The selective detection of *Salmonella* was due to the use of anti-*Salmonella* polyclonal antibodies covalently immobilized onto the quantum dot surface. *Salmonella* cells were extracted from the sample and concentrated using superparamagnetic particles and a microfluidic chip. The same team [140] developed a portable spectrofluorometer which was able to measure the fluorescence signal emitted by the quantum dot nanoparticles linked to *Salmonella* cells. A correlation between the fluorescence response of the sensor and the *Salmonella* Typhimurium cell concentration was obtained and the detection limit was evaluated in borate buffer and chicken extract at 10^3^ CFU/mL (Table 2).

#### 2.1.3. Chemical Luminescence-Based Biosensors

Chemical luminescence biosensors are based on the measurement the light emitted during reactions of bio-chemiluminescence, thermochemiluminescence, or electrogenerated chemiluminescence [156]. Their major advantage is related to the high detectability of the light during the chemical reaction without nonspecific signal.

Recently, Oh and Park [132] validated the use of immunosensors associated with a light microscopic imaging system (LMIS) for the fast detection of *Salmonella* in chicken. The authors obtained a LOD of 10^3^ CFU/25 g of chicken skin, which is lower than those of other reported sensors [132]. Immunosensors combined with LMIS allowed a direct observation and enumeration of *Salmonella* (Table 2).

**Figure 5:**
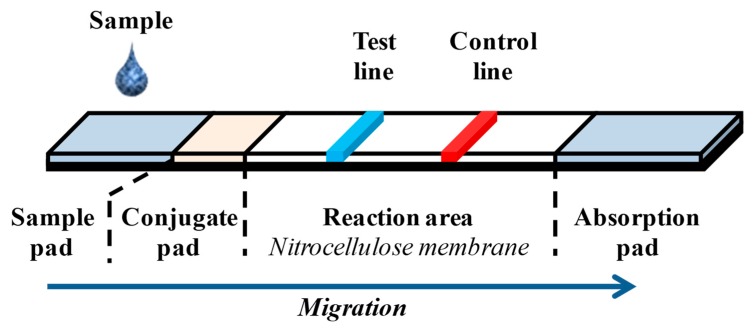
Structure of the lateral flow assay system.

Current researches are focused on the development of low cost and rapid detection techniques, with less sample treatments and less technical expertise but with high specificity. Lateral flow assays (LFA) meet these criteria in terms of simplicity, rapidity, high specificity, sensitivity, versatility, and long-term stability under different environmental conditions. LFA provides a good alternative to realize qualitative and quantitative analysis and its advantages and disadvantages are presented in Table 3 [157]. LFA have been developed and used for the analysis of hormones, heavy metals, bacteria, virus, and toxins into variable matrices like human or animal fluids, food, water, and environment. Classical LFA are performed over a strip, the different parts of which are arranged such that they overlap each other on a plastic backing (Figure 5). These different parts are composed of a sample application pad, a conjugate pad, a nitrocellulose membrane, and an adsorption pad at the end. The nitrocellulose membrane is divided into test and control lines (Figure 5). All the reagents are immobilized in the different parts of the strip and are activated during the migration of the liquid sample. A lot of LFA have been reported for the detection of bacterial pathogens in various sample matrices. Different types of colored revelation reagents can be used in LFA, for example, quantum dots (QDs), carbon nanotubes (CNTs), magnetic particles (MPs), enzymes, gold nanoparticles (AuNPs), and colored latex beads. Unipath commercialized the first LFA test named "Clearblue" for home pregnancy tests. This test was based on the use of blue dye-doped latex particles. However, colloidal gold nanoparticles (AuNPs) are still the most currently used as a label because of their easy synthesis, visual detection, and stability [157,158,159,160,161]. Recently, Xia et al. [47] developed an immunological LFA for the detection of entire cells of *Salmonella* Choleraesuis (*S*. Choleraesuis), using gold magnetic bifunctional nanobeads as label. A sensitivity of 5 × 10^5^ CFU/mL was obtained. This LFA was applied to the detection of *S*. Choleraesuis in whole milk and the results were obtained after 20 h cultivation in selective medium (Table 2). Another immunochromatographic assay was developed for the detection of *S*. Typhimurium and Enteritidis in a single chip [145]. The assay was based on a sandwich format involving two specific anti-*S*. Typhimurium and anti-*S*. Enteritidis antibodies immobilized on a nitrocellulose membrane at separated test lines, while the other specific antibody to *Salmonella* spp. was conjugated with gold nanoparticles. This LFA detected *S*. Typhimurium and *S*. Enteritidis in culture medium at concentrations of 10^4^ and 10^6^ CFU/mL, respectively. Further analyses of spiked chicken samples showed a specificity of 100% for the two *Salmonella* serovars [145] (Table 2). More recently Viter et al. [141] developed an optical biosensor for the detection of *Salmonella* Typhimurium based on the photoluminescence of TiO_2_ nanoparticles. Antibodies directed against *Salmonella* surface antigens were immobilized on the surface of a glass coated with nanoparticles of titanium dioxide (TiO_2_). At room temperature, the TiO_2_ nanoparticles exhibited an intense photoluminescence (PL) in the visible range, which was modified upon *Salmonella* cells binding. This immunosensor allowed the detection of *Salmonella* in the range 10^3^ to 10^5^ cell/mL.

### 2.2. Electrochemical Biosensors

Electrochemical detection methods are increasingly used for identification and quantification of food borne pathogens due to several advantages such as rapidity, ease of use, cost effectiveness, and easy miniaturization. Electrochemical biosensors are classified into amperometric, voltammetric, potentiometric, impedimetric, and conductimetric, based on the measured parameters such as current, potential, impedance, and conductance, respectively. The electrodes used as transducers may be modified to improve the performance of the sensors, for example, by the conjugation of specific recognition elements (antibodies, oligonucleotides, etc.) to increase the specificity of the detection, or by the introduction of nanomaterials (carbon nanotubes, etc.) to increase the measured signal, and therefore decrease the LOD.

#### 2.2.1. Amperometry

Amperometry is an electrochemical technique that allows the detection of electroactive compounds. It is based on the measurement of a current intensity at a fixed potential.

Abdel-Hamid et al. [46] developed a flow-injection amperometric immunofiltration assay for the rapid detection of total *E. coli* and *S*. Typhimurium. A flow injection cartridge composed at the top of a porous nylon membrane was used as a support for the immobilization of anti-*E. coli* or anti- *Salmonella* capture antibodies. The working electrode, the reference electrode, and the counter electrode were positioned downstream. The liquid sample was injected through the cartridge and the immobilized antibody captured the bacterial. Peroxidase-labeled antibodies were then injected and bound to captured bacteria. After the addition of the enzyme substrate, an amperometric signal was recorded which was generated by reduction of enzymatic product. This immunofiltration system which was based on a sandwich immunoassay scheme was able to specifically and directly detect 50 to 200 cells/mL of *E. coli* or *S*. Typhimurium in 35 minutes, with a detection limit of 50 cells/mL for *E. coli* or *S*. Typhimurium (Table 2).

#### 2.2.2. Potentiometry

Potentiometry is one of the most common, cheap, simple and portable electrochemical technique. It is based on the measure of a change of potential between two electrodes.

Dill et al. [146] were able to detect *S*. Typhimurium in carcass washing samples at a level as low as 119 CFU within 15 minutes using the Threshold^®^ immunoassay system. In a first step, the analyte (*Salmonella*), a biotinylated anti-*Salmonella* antibody, a fluorescein-labeled anti-*Salmonella* antibody, and streptavidin were mixed to form an immunocomplex in solution phase. The two antibodies were necessary for the formation of analyte-immunosandwich complex. In a second step, this immunocomplex was filtered through a 0.45 μm biotin-coated microporous nitrocellulose membrane, leading to immobilization via biotin-avidin affinity. Finally, the binding of *Salmonella*-specific immunocomplex was revealed by the addition of a urease-bound antifluorescein antibody. In presence of urea, the urease converted the substrate into ammoniac and CO_2_, inducing a variation of pH at the surface of the chip. The resultant pH change was monitored with time and the output signal is reported in µV/s (Table 2).

#### 2.2.3. Impedimetry

Electrochemical impedance spectroscopy (EIS) is an effective technique for sensing the binding of compounds onto the surface of an electrode by measuring the characteristics of the electrode and electrolyte interfacial properties, and more specifically the charge transfer resistance. EIS is especially suited for the development of affinity-based biosensors as it allows the label-free detection of binding events [133].

For the first time, Mutreja et al. [147] developed an impedimetric immunosensor based on the use of a specific surface antigen, OmpD, as a biomarker for the detection of *S*. Typhimurium. Anti-OmpD antibodies were used as detector probe to develop an immunoassay on graphen oxide modified screen-printed carbon electrodes. Some water samples were artificially contaminated with *S*. Typhimurium cells, and the impedance response was studied. The resulting immunosensor was able to detect *S*. Typhimurium with a sensitivity of 10^1^ CFU/mL [147] (Table 2). Another impedimetric biosensor for *S*. Enteritidis detection was developed by Kim et al. [148] that detected the impedance variation caused by the attachment of *Salmonella* cells onto corresponding antibodies immobilized on interdigitated gold electrodes. This biosensor was able to detect *S*. Enteritidis cells at a level of 10^6^ CFU/mL in buffer within three minutes. The detection performances were enhanced by the use of nanoparticles. Using nanoparticles, the sensor performances were greatly enhanced, with detection limits of 10^4^ CFU/mL and 10^5^ CFU/mL of *S*. Enteritidis in buffer and milk, respectively (Table 2). Another team used the nanoparticles [149] to develop an impedance immunosensor which rapidly and sensitively detected *Salmonella* Typhi (*S*. Typhi) in small volume sample (10 µL). The cells were tagged with gold nanoparticles via high-affinity antigen–antibody interactions and micron-gap interdigitated electrodes were used to generate high electric field gradients near the electrode edges to improve the signal collection efficiency. The signal from the linked gold nanoparticles was used for the quantification of the cells present in the 10 µL of sample loaded into the sensor. The assay was achieved in 5 minutes and a LOD of 100 CFU/mL was obtained (Table 2). More recently, an impedimetric biosensor was developed by Guler Gokce et al. where a capture DNA probe was covalently immobilized onto polyurethane/poly(m-anthranilic acid) (PU/P3ANA) nanofibers [144]. Both immobilization and hybridization processes were investigated by electrochemical impedance spectroscopy (EIS). The resulting DNA biosensor showed a linear response for DNA concentrations ranging from 0.1 µM to 10 µM and a high sensitivity (8.17 kΩ/µM) (Table 2). The sensor was selective to a single-base mismatch and was stable up to one month.

### 2.3. Mass-Based Biosensors

The transduction of mass-based biosensors consists of measuring the oscillation induced by small mass variations on a piezoelectric crystal surface. Bulk wave (BW) or quartz crystal microbalance (QCM), and surface acoustic wave (SAW) represent the two main types of mass-based sensors. Mass-based detection often allows direct label-free analysis, with good sensitivity and high specificity.

Piezoelectric sensors for bacterial detection are generally coated with appropriate antibodies and the sensor is directly introduced in the solution containing the target cells. When the cells bind antibodies, the mass increase at crystal surface induces a proportional oscillation decrease. According to the Sauerbrey equation, the frequency decrease is proportional to the mass change, which depends on bacterial concentration.

Prusak-Sochaczewski et al. [150] developed a QCM biosensor for the detection of *S*. Typhimurium. The antibodies grafted on the crystal were selective for a structural antigen present in a large number of *Salmonella* species. The sensor had a linear response between 10^5^–10^9^ cells/mL of microbial suspension. It was shown that 5 h are required to analyze a sample at 10^5^ cells/mL of *S*. Typhimurium. According to the authors, the coated crystal was stable for six to seven days and the crystal could be reused if the bacteria were removed using concentrated urea (8M) (Table 2). König et al. [151] used a similar sensor for the detection of *Salmonella*, among other bacteria. The resulting sensor showed a linear response in the range of 10^6^–10^8^ cells and, according to the authors, the sensor could be reused 12 times (Table 2). The detection of *S*. Typhimurium was also described using a piezoelectric biosensor in a flow-injection system [152]. In this case, anti-*Salmonella* spp. antibodies were immobilized onto a gold quartz crystal surface using the polyethylenimine-glutaraldehyde (PEG) coupling technique. The results were obtained in 25 minutes with a linear range of 5.3 × 10^5^ to 1.2 × 10^9^ CFU/mL (Table 2). Other authors used the Langmuir–Blodgett method to immobilize polyclonal antibodies against *S*. Typhimurium on the surface of a quartz crystal acoustic wave device [153]. This sensor showed a detection limit of a few hundred cells/mL, a response time under 100s, a working range of 10^2^–10^10^ cells/mL, and a linear response between 10^2^–10^7^ cells/mL. According to the authors, the sensors preserved 75% of their sensitivity over a period of 32 days (Table 2). Su et al. [154] developed a QCM immunosensor which allow the detection of *S*. Typhimurium by simultaneously measuring resonance frequency and motional resistance. The antibodies were immobilized onto the crystal gold surface via protein A interaction. When analyzing chicken meat samples, the best results were obtained by measuring resonance frequency, which was proportional to the *S*. Typhimurium concentration in the range of 10^5^–10^8^ cells/mL. The detection limit was even decreased to 10^2^ cells/mL by using anti-*Salmonella*-modified magnetic beads as a separator/concentrator agent during sample pretreatment (Table 2). Recently, a QCM instrument with a microfluidic system for the rapid and real-time detection of *S*. Typhimurium was developed by Salam et al. [155]. A gold sensor chip with two sensing areas was modified by carbodiimide to allow the grafting of the anti-*Salmonella* monoclonal antibody (capture antibody) on the active spot and the grafting of a mouse IgG antibody on the control spot. The recognition of *Salmonella* cells by immobilized anti-*Salmonella* antibodies induced a change in frequency of the quartz crystal resonator that was correlated to *Salmonella* concentration. *Salmonella* cells were detected using either direct, sandwich, or sandwich assay with antibody-conjugated gold nanoparticles. The highest sensitivity was obtained using gold nanoparticles modified antibodies, with a LOD near 10–20 CFU/mL Direct and sandwich assays had detection limits of 1.83 × 10^2^ CFU/mL and 1.01 × 10^2^ CFU/mL, respectively. This sensor, developed by Salam et al., has shown good sensitivity and selectivity against *Salmonella* despite the presence of endogenous bacteria in chicken meat samples (Table 2).

Despite these good performances, it must be stressed that detection and quantification of bacteria using piezoelectric sensors may be a relatively long process in terms of incubation time and due to the numerous washing and drying steps required before the measurement.

## 3. Aptasensors for *Salmonella* Detection

When used as the molecular recognition elements, antibodies allow a direct and rapid detection of *Salmonella* with high affinity and specificity. Despite these advantages, antibodies have several drawbacks related to their laborious production and their relative instability [162]. To overcome the limitations encountered with these immunoreagents, the development of alternative affinity molecules, such as nucleic acids (DNA or RNA), has appeared as a promising solution. Classical nucleic acid biosensors, also called genosensors, allow the detection of target specific genes, they are based on the hybridization of a single-strand DNA (ssDNA) to its complementary strand. The strategy based on DNA complementarity has already been exploited in DNA microarrays for several applications including gene expression analysis, polymorphism studies, and genotyping [7,25,119,163,164,165]. In contrast, aptamers are short oligonucleotides that are able to adopt a stable secondary structure capable of binding a target molecule with high affinity and specificity. Due to their remarkable properties, aptamers have become highly attractive for developing analytical tools such as biosensors for food analysis, environment monitoring, and medical diagnostic [166,167]. As aptamers can be designed for a great variety of targets, ranging for ions to whole cells, several aptasensors have already been described for the detection of various microorganisms [168,169,170].

### 3.1. Aptamers Selection

Aptamers are short DNA or RNA molecules that can bind with high affinity and specificity to their target molecules, which can be drugs, proteins, toxins, sugar, antibiotics, and bacteria. However, RNA aptamers appeared to be less stable than DNA aptamers. The synthesis of aptamers presents many advantages compared to the production of antibodies as it is fast, affordable, does not involve animal production, and does not suffer from batch-to-batch variations. DNA aptamers are stable over time are they are resistant to high temperatures, they generally show a high affinity for their target, and they can be easily modified by chemical groups for immobilization or labeling purposes.

**Figure 6:**
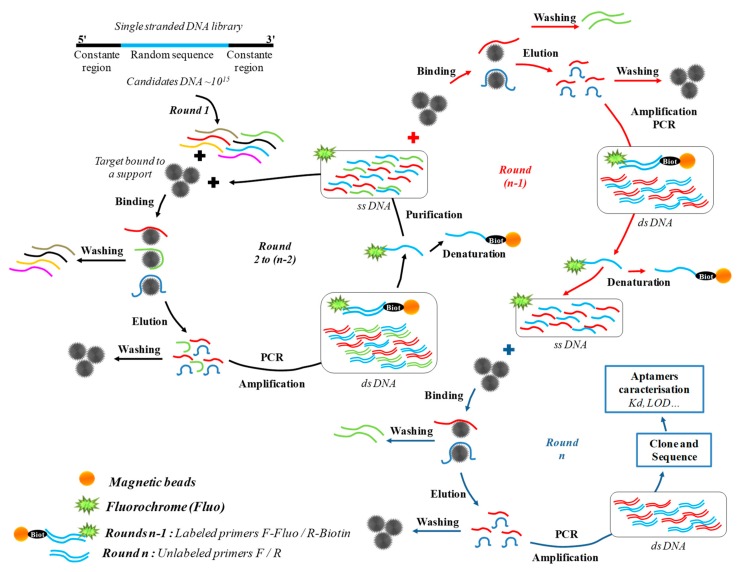
Synoptic representation of the SELEX method for DNA library. Three main stages constitute a general SELEX protocol, the incubation of the library with the target which is sometimes bound to a support, the separation of the oligonucleotides linked to the target from the unbound oligonucleotides from the library, and the amplification of the oligonucleotides linked to the target. For this representation, the primers are modified with a fluorochrome for the forward primer and with biotinylated magnetic beads. After the last stage, the amplified oligonucleotides (dsDNA) are denatured, in this representation, with the help of magnetic beads which are retained by a magnet, and therefore only the ssDNA tagged with the fluorochrome are used for the next round. The presence of the fluorochrome tracks the amounts of aptamers selected during the SELEX process. For RNA-SELEX, some additional steps are included first, i.e., an in vitro transcription to obtain an RNA library, and the reverse transcription of bound RNA molecules to obtain cDNA and its subsequent amplification.

Aptamers are primarily selected through an entirely in vitro combinatorial biochemistry method, called systematic evolution of ligands by EXponential enrichment (SELEX) (Figure 6). This process consists of bringing the target molecule into contact with a single-stranded random oligonucleotide library of DNA or RNA (approximately 10^15^ candidates). After partitioning and discarding the non-binding oligonucleotides, the target-bound candidates are amplified and used in the next selection round. Consecutive binding selection-amplification steps are repeated seven- to 15-fold, leading to the selection of the more specific candidates [25] (Figure 6), which are then cloned and sequenced to identify the consensus motif corresponding to the minimal sequence capable of highly specific binding of the target [171] (Figure 6). The classical SELEX procedure has been already used for selecting aptamers against a wide range of pathogenic microorganisms [25,172]. These aptamers are able to bind to cell surface proteins, for example, the PilS protein of *S. enterica* type IVB pili [173]. In the same manner, Joshi et al. [172] selected one aptamer targeting the outer membrane proteins (OMPs) of *S*. Typhimurium. However, the classical SELEX method that can be used for purified and soluble protein targets is often not adapted to membrane proteins as it requires the presence of cell membrane or a co-receptor to fold correctly. For this reason, some alternative SELEX methods were developed where a live whole cell is used as a target. This method, called whole-cell SELEX, was realized for the first time by Bruno et al. [174] for the selection of DNA aptamers against the spores of a non-pathogenic *Bacillus anthracis* strain. Since then, the whole cell-SELEX process has been applied by many teams for the selection of aptamers against the food pathogen strains of the genus *Salmonella*. The whole cell-SELEX was used for the selection of two DNA aptamers against *S*. Enteritidis and *S*. Typhimurium, and more particularly against these bacteria in VBNC state [175,176]. These two cell-SELEX were conducted in twelve rounds of selection with a positive selection step against viable *S*. Enteritidis and *S*. Typhimurium. A negative selection step was added with a mixture of related pathogens including *E. coli*, *S. aureus*, *P. aeruginosa*, and *Citrobacter freundii* with *S*. Typhimurium or *S*. Enteritidis. After sequencing, two aptamers were retained for their high affinity to *S*. Enteritidis and *S*. Typhimurium strains (Table 4). Another team [177] selected a DNA aptamer for *S*. Typhimurium by cell-SELEX with a relatively high binding affinity and with a dissociation constant (Kd) of 1.73 ± 0.54 μM (Table 4). Similarly, Duan et al. [178] isolated another DNA aptamer for the detection of *S*. Typhimurium with a Kd of 6.33 ± 0.58 µM (Table 4). A DNA aptamer for *S*. Typhimurium was also obtained after 10 rounds of selection and counter selection with a mix of *S*. Enteritidis, *E. coli*, and *S. aureus* [179]. Recently, a study focused on the selection of DNA aptamers toward live cells of *S*. Enteritidis and *S*. Typhimurium by whole cell-SELEX. After 10 rounds of selection and a counter selection during the seventh round with a mixture of *S*. Enteritidis, *S*. Typhimurium, *P. aeruginosa*, and *E. coli*, two DNA aptamers for *S*. Enteritidis and one for *S*. Typhimurium were selected [180]. The two aptamers for *S*. Enteritidis showed Kd values of 4.66 μM and 3.8 μM, while the aptamer for *S*. Typhimurium had a Kd of 0.530 μM (Table 4). RNA aptamers have also been described for the detection of *S*. Typhi [173] and *S*. Enteritidis [181] using classical SELEX with PiLS protein as target and the whole cell-SELEX protocol (Table 4).

In conclusion, whole cell-SELEX appears as a very efficient procedure for the selection of aptamers against pathogens, as it does not need any isolation and purification of the target component. Moreover, the aptamers are selected when the targets are in their native conformation on the cell surface, so that they are able to recognize the whole bacteria with a higher specificity, by sometimes binding several sites of the cell membrane. In Table 4, the different aptamers developed against different *Salmonella* species and serovar are summarized and, as can be seen from the table, Kd value for most of the aptamers ranges from 7 × 10^−3^ to 4.6 µM, demonstrating their good affinity.

### 3.2. Aptamers as Ligands for Magnetic Separation

Due to their high affinity, the aptamers can be used as a ligand to realize a magnetic capture or separation of the target from its matrix. For example, an aptamer (S8-7) selected by whole cell-SELEX (Table 4) was successfully used for the magnetic capture of *S*. Typhimurium cells in buffer [177]. The strain capture was followed by qPCR detection. The LOD of the aptamer magnetic capture qPCR assay was from 10^2^ to 10^3^ CFU, equivalent to *S*. Typhimurium in 290 µL of sample [177]. This study provides proof-of-concept that biotinylated aptamers selected by whole cell-SELEX method can be used in a qPCR-based capture-detection platform dedicated to *S*. Typhimurium. Another example is the selection of DNA aptamers against the outer membrane proteins (OMPs) of *S*. Typhimurium [172] (Table 4). The aptamer, named 33, was selected and bound to magnetic beads to allow the capture of *S*. Typhimurium into whole carcass chicken rinse samples. *S*. Typhimurium extracted from the matrix were detected and quantified by using real-time PCR. The same team showed interesting results with detection limits of 10^1^ to 10^2^ CFU of *S*. Typhimurium for 9 mL of rinsate in a pull-down assay format, and detection limits of 10^2^ to 10^3^ CFU in 25 mL of rinsate in recirculation format [172].

### 3.3. Optical Aptasensors

According to the used transducers, four types of aptasensors have been described for *Salmonella* detection, involving surface plasmon resonance (SPR), surface-enhanced Raman (SER), and fluorescence or chemiluminescence detection [188].

#### 3.3.1. Surface Plasmon Resonance Aptasensors

As described before, SPR spectroscopy is a mass-sensitive sensor that detects the mass change. When the incident light at a critical angle of incidence enters into the resonator with two different refractive indexes, it leads to resonation of the electrons of the metal [188]. SPR aptasensors are label-free, can be miniaturized to become portative, and the analysis can be easily automated.

Recently, Yoo et al. [189] developed a single localized surface plasmon resonance (LSPR) sensor for the detection and identification of three different bacterial species, including *S*. Typhimurium. This system was based on a multispot gold-capped nanoparticles array chip composed of a dielectric layer comprised of a thin gold layer on a silica nanoparticles-absorbed glass slide. Each species-specific aptamer was immobilized on each spot of the chip. For *S*. Typhimurium the authors used the aptamer sequence already obtained by Joshi et al. [172] (Table 4) and the resulting aptasensor showed a detection limit of 30 CFU/mL (Table 5).

#### 3.3.2. Surface-Enhanced Raman Spectroscopy Aptasensors

Surface-enhanced Raman spectroscopy (SERS) is a surface-sensitive technique that enhances Raman scattering by molecules adsorbed on rough metal surfaces or nanoparticles [188]. When the light gets through the media, the incident photons and molecules collide with each other and the molecular vibrational/rotational energy and photon energy superimpose, producing the scattering spectrum due to the change of frequency [188].

Ravindranath et al. [190] developed a SERS aptasensor that allows the simultaneous detection of *S*. Typhimurium, *S. aureus*, and *E. coli* O157:H7. An aptamer for *S*. Typhimurium and two antibodies for *S. aureus* and *E. coli* were immobilized onto gold, silver, and silver-gold core-shell nanoparticles, respectively, labeled with Raman dye molecules. The results showed a good specificity and sensitivity of the SERS aptasensor that simultaneously detected the three bacteria within 45 minutes, with a detection limit of 10^2^ CFU/mL (Table 5).

#### 3.3.3. Chemiluminescent Aptasensors

Chemiluminescence corresponds to the light radiation produced by a particular kind of molecule that are able to adsorb chemical energy. This method had a high sensitivity, is simple, inexpensive, and can easily be miniaturize.

Recently, Yang et al. [184] used the SELEX method to isolate a DNA aptamer against *S*. Paratyphi A, which was called Apt22 (Table 4). The authors designed a detection probe (P0) by melting Apt22 sequence (P2) with a horseradish peroxidase mimicking DNAzyme (P1). P0 was allowed to bind by noncovalent self-assembly with single-walled carbon nanotubes (SWNTs). When the targets, *S*. Paratyphi A and hemin, were added, they bind to the P2 and P1 sequences, respectively, resulting in P0 dissociation from SWNTs and formation of an active hemin/G-quadruplex DNAzyme. The liberated DNAzyme then act as a catalyst for the generation of chemiluminescence signal through the oxidation of luminol by H_2_O_2_. This detection system was validated for the detection of *S*. Paratyphi A in artificially contaminated city water samples with a detection limit of 10^4^ CFU/mL (Table 5).

#### 3.3.4. Fluorescent Aptasensors

Fluorescent aptasensors are mainly based on the fluorescence polarization or fluorescence intensity change produced by the interaction of targets and fluorescent probe labeled aptamers [188]. A fluorescent bioassay was developed in 2012 for the simultaneous detection of *S*. Typhimurium and *S. aureus* using two aptamers immobilized on magnetic nanoparticles (Table 4) [191]. The secondary modification of nanoparticles with NaYF_4_,Yb, Er/Tm, allowed the emission of a luminescent signal when the complexes were laser-excited at 980 nm. This luminescent signal was amplified by the magnetic separation and concentration. A LOD of 5 CFU/mL was obtained for *S*. Typhimurium (Table 5) [191]. In another study, Duan et al. [178] selected an aptamer (ST2P) against *S*. Typhimurium by the whole bacterium-based SELEX method (Table 4). Some ST2P aptamers were conjugated to magnetic nanoparticles for capture purpose, while others where labeled with a fluorescent dye (FAM) for detection purpose. As several copies of each type of aptamer were able to recognize and bind the bacterial strain, the quantification of *S*. Typhimurium was possible after a simple magnetic separation, and the observed LOD was 25 CFU/mL (Table 5). Using the same aptamer sequence, Duan et al. [192] developed a flow cytometry bioassay for *S*. Typhimurium using quantum dots (QDs) as fluorescent markers (Table 4 and Table 5). The detection limit of this bioassay was 5 × 10^3^ CFU/mL for *S*. Typhimurium. To overcome the use of complex analytic equipment, such as cytometers, some authors used nanogold particles as markers, allowing a naked eye reading or a simple colorimeter to obtain the result. Yuan et al. [7] developed a sandwich assay complex using a capture aptamer immobilized on microplate wells, allowing bacterium recognition, a revelation aptamer coupled with gold nanoparticles (AuNPs), and silver staining amplification (Table 4). The described bioassay had a LOD of 7 CFU/mL (Table 5).

A similar bioassay was described using label-free aptamers that adsorbed on the surface of unmodified AuNPs for the detection of *E. coli* O157:H7 and *S*. Typhimurium [185] (Table 4). The detection was carried out by the aggregation of the AuNPs induced by the presence of the target bacteria, which was associated with a red-to-purple color change upon high-salt conditions. This system allowed detecting as low as 10^5^ CFU/mL *S*. Typhimurium, within 20 min and a specificity of 100% (Table 5). More recently, Wang et al. [193] developed a sandwich-type fluorescent aptameric assay allowing the simultaneous detection of *S. aureus* and *S*. Typhimurium, based on the aptamers described by Joshi et al. [172] (Table 4). Signal probes consisted of the aptamers labeled with multi-color lanthanide-doped time-resolved fluorescence nanoparticles, while aptamers immobilized on Fe_3_O_4_ magnetic nanoparticles were used as capture probes. Due to the magnetic separation and concentration of Fe_3_O_4_ nanoparticles, detection limits were 15 CFU/mL (Table 5).

#### 3.3.5. Colorimetry-Based Aptasensors

Bayraç et al. [196] developed a sandwich-type aptamer-based colorimetric platforms were the aptamer against *S*. Enteritidis was selected by cell systematic evolution of ligands by EXponential enrichment (cell-SELEX). The authors selected two aptamers with a Kd of 0.971 and 0.309 μM after 12 rounds of cell-SELEX. The two aptamers were used to develop two sandwich-type capillary detection platforms, where the detection of the bacteria was based on color change visible to the naked eye. For the two aptamers the detection limit was of 10^3^ CFU/mL in cell suspension and milk samples [196].

#### 3.3.6. Flat Substrate Aptasensors

The detection limit has been decreased to 10^4^ cells by silver enhancement. The team of Fang, developed a particular lateral flow assay for the detection and quantification of *Salmonella* Enteritidis [131] (Table 4). For the detection, two aptamers against different outer membrane proteins of *S*. Enteritidis were used. One of the aptamers was used to realize the magnetic bead enrichment and the second was used as a template to carry up a strand displacement amplification (SDA). Finally, the single-strand DNA obtained by SDA was detected with a lateral flow biosensor. The LOD of this bioassay was 10^1^ CFU of *S*. Enteritidis (Table 5).

### 3.4. Electrochemical Aptasensors

Electrochemical aptasensors constitute the immobilization of the aptamers (DNA or RNA) onto the electrodes surfaces with or without a second element, which add an electrochemical activity. In the presence of the target, a change in the structure of the aptamers on the electrode surface occurs, that induced a variation in the electrochemical signal (current, impedance, potential, or conductance). This variation has been analyzed and the result was correlated with the target concentration or with the presence of the target in the case of an on/off biosensor.

Electrochemical aptasensors combine the high specificity of the recognition between the target and the aptamer with the high sensitivity of the electrochemical biosensors.

#### 3.4.1. Potentiometry

Zelada-Guillén et al. [102] developed a label-free potentiometric aptasensor for detecting *S*. Typhi (Table 4). The aptamer sequence was modified with a five-carbon spacer and an amine group at the 3’ end and was covalently immobilized into a layer of carboxylated single-walled carbon nanotubes (SWCNTs) by a π–π stacking interaction [197]. The couple aptamer SWCNT corresponds to the sensing and the transducing layer of the sensor. When the target was absent, the aptamers were self-assembled on carbon nanotubes by the π–π stacking interaction. In presence of the target, *S*. Typhi, a conformational change in the aptamer appeared and the phosphate groups were separated from the SWCNT that induced a charge change and a variation of the recorded potential. This biosensor allowed the detection of *S*. Typhi, in phosphate buffer, from 0.2 CFU/mL to 10^6^ CFU/mL in a short response time of 60 s (Table 5).

#### 3.4.2. Impedimetry

An aptamer-based impedimetric sensor for the typing of bacteria (AIST-B) in particular *S*. Enteritidis was developed by Labib et al. [176] (Table 4). First, DNA aptamers were selected by cell-SELEX technique after twelve rounds of selection. The most specific aptamer with the best binding affinity to *S*. Enteritidis was used for the development of an impedimetric sensor via self-assembly onto gold nanoparticles-modified screen-printed carbon electrode. Their aptasensor could detect 18 cells of *S*. Enteritidis in 30 µL (600 cells/mL) in 10 min and was able to distinguish *S*. Enteritidis from the other species *S*. Typhimurium and *S*. Choleraesuis (Table 5). The team of Labib also developed an aptamer-based sensor designed for the detection of live cells and also for the detection of VBNC cells of *S*. Typhimurium [175] (Table 4). First, a highly specific DNA aptamer against *S*. Typhimurium was selected by cell-SELEX technique after twelve rounds of selection and a sequencing step. Finally, the DNA sequence with high binding affinity was integrated onto gold nanoparticles-modified screen-printed carbon electrode to develop an impedimetric sensor. Their aptamer-based viability impedimetric sensor (AptaVISens-B) was able to detect at least 18 live cells in 30 µL of sample (600 cells/mL) and was able to distinguish live and heat killed *S*. Typhimurium (Table 5). Some researchers developed some impedimetric biosensors with a better LOD. Ma et al. [194] developed an electrochemical biosensor based on a glassy carbon electrode modified with graphene oxide and ssDNA aptamer against *Salmonella* linked on gold nanoparticles for the specific detection of *Salmonella* genus. They used the aptamer sequence previously obtained by Joshi [172] (Table 4). The *Salmonella* cells were incubated on the modified electrode and the electrochemical impedance spectrum was measured. This aptamer-based electrochemical biosensor had a linear relationship between 2.4 CFU/mL and 2.4 × 10^3^ CFU/mL and a detection limit of 3 CFU/mL (Table 5). Another team developed an impedimetric biosensor with a modified electrode were a copolymer, the poly [pyrrole-co-3-carboxyl-pyrrole], was on the surface of the electrode and the aptamer were grafted on the polymer [186] (Table 4). This label-free electrochemical biosensor was suitable for the detection of *S*. Typhimurium in the concentration range of 10^2^ to 10^8^ CFU/mL with a LOQ of 100 CFU/mL and a LOD of 3 CFU/mL (Table 5). Recently, another label-free impedimetric aptamer-based biosensor for *S*. Typhimurium detection was developed by Bagheryan et al. [187] (Table 5). This biosensor was designed by grafting a diazonium supporting layer onto screen printed carbon electrodes and by the chemical immobilization of the aminated aptamer obtained from the work of Joshi et al. [172] (Table 4). This impedimetric aptasensor had a linear respond, on a logarithm scale from 10^1^ to 10^8^ CFU/mL with a LOQ of 10^1^ CFU/mL and a LOD of 6 CFU/mL. Their aptasensor was able to discriminate *S*. Typhimurium from six other bacteria strains. They also validated the ability of the biosensor to detect *S*. Typhimurium in artificially contaminated apple juice samples at concentrations of 10^2^, 10^4^, and 10^6^ CFU/mL.

More recently, Ranjbar et al. [182] developed an electrochemical aptasensor based on the use of nanoporous gold as a substrate for *S*. Typhimurium detection. A thiol functionalized aptamer against *S*. Typhimurium (Table 4) was linked to the surface of NPG/Au/GCE via self-assemble monolayers (SAMs) formation. Using EIS, this aptasensor was capable of detecting *S*. Typhimurium in a wide linear dynamic range 6.5 × 10^2^ to 6.5 × 10^8^ CFU/mL with a LOQ of 6.5 × 10^1^ CFU/mL and LOD of 1 CFU/mL, and was able to distinguish live cells from dead [182]. The biosensor was tested with real samples. Eggs were spiked with different amounts of *Salmonella* (6.5 × 10^3^ to 6.5 × 10^7^ CFU/mL) and the recovery of the sensor was comprised between 84.61% and 109.07%. The results were obtained in 40 min [182].

#### 3.4.3. Differential Pulse Voltammetry (DPV)

Dinshaw et al. [183] developed an electrochemical aptasensor using electrochemically-reduced graphene oxide-chitosan (rGO-CHI) composite as a conductive substrate for the detection of *Salmonella* enterica serovar Typhimurium whole cell. The biorecognition element of this aptasensor was a thiol-functionalized aptamer against the outer membrane protein of *Salmonella* (Table 4) immobilized on rGO-CHI by using glutaraldehyde as the crosslinker. The aptasensor exhibited a low LOD of 10^1^ CFU/mL for *S*. Typhimurium. They have also tested the aptasensor with artificially spiked raw chicken samples and the results were in line with the results obtained with pure cultures. This aptasensor was specific to *Salmonella* and could distinguish between *Salmonella* enterica cells and non-*Salmonella* bacteria (*S. aureus*, *K. pneumonia*, and *E. coli*).

### 3.5. Mass-Based Aptasensors

Quartz crystal microbalance aptasensors are based on the immobilization of the aptamer on the quartz crystal. When the aptamer recognizes and retains the target, the load on the surface of the quartz increases and the oscillation frequency of the quartz varies and is correlated to the mass adsorbed on the quartz. This aptasensor is simple and has a high sensitivity.

Ozalp reported on a sensitive strategy for the detection of *S*. Typhimurium cells in food samples based on the combination of an aptamer-based magnetic separation system, for a rapid enrichment of target pathogens, and a QCM analysis for specific and real-time monitoring [195] (Table 4). The system could capture *S*. Typhimurium cells in 10 min from milk samples and the QCM allowed the specific detection of the strain. A linear response ranging from 100 to 4 × 10^4^ CFU/mL cells was obtained, as well as a LOD of 100 CFU/mL (Table 5). Moreover, the aptamer sensor can be regenerated by the addition of a NaOH solution at the surface of the QCM crystal.

## 4. Conclusions

The common strategies for the detection of the foodborne pathogen *Salmonella* spp. consist of the gold standard conventional microbiological culturing techniques, PCR methodologies, and immunology techniques. In addition to these methods, the development of novel strategies involving biosensors and, more particularly, aptasensors is a real alternative for the rapid and low-cost detection of foodborne bacteria. Now, aptamers can be easily selected using SELEX technology that includes a variety of techniques that are flexible and tunable enough to target any compound of interest. The high selectivity of aptamers has allowed the development of sensitive and selective aptasensors for the determination of *Salmonella* spp. strains (Table 2 and Table 5) in various food matrices. All these researches for the selection of new aptamers against *Salmonella* spp., as well as the promising results achieved for the detection of *Salmonella*, show that aptamer-based technologies could become a real alternative to conventional strategies for the detection of other foodborne pathogens.

## Figures and Tables

**Table 1:** Advantages and drawbacks of the conventional methods used for the detection of *Salmonella* in food.

	Culture and Colony-Based Methods	Immunology Based Methods	Polymerase Chain Reaction	DNA Based Methods
**Advantages**	Low coast Sensitivity Selectivity with chromogenic media	Fast Robust Specificity “Real time” analyses	Specific Sensitive Rapid Accuracy Detection of small amounts of target nucleic acid	Specific Sensitive Rapid Reusability Stability Detection of small amounts of target
**Drawbacks**	Labor intensiveness Time-consuming Low sensitivity Microbial contamination VBNC	Low sensitivity Low affinity of the antibody to the pathogen or other analyte Interference from contaminants	False negative PCR results No distinction between dead or alive cells	No distinction between dead or alive cells
**Progress**	Association with DNA, antibody, or biochemical-based methods	Association with other methods: Immunomagnetic separation on magnetic beads coupled with matrix-assisted laser desorption ionization time-of-flight mass spectrometry, combination of immunomagnetic separation with flow cytometry	Reverse Transcriptase PCR (RT-PCR) to distinguish live and dead cells Association with another method, the biosensors	Design of aptamers

**Table 2:** Biosensors for *Salmonella* strains detection in food samples.

Microorganism	Sample Matrix	Bioreceptor	Immobilization Method	Transducer	Limit of Detection	Analyze Time	Working Range	References
*S*. Typhimurium	Chicken carcass	Antibody to Common Structural Antigens (CSA-1)	Succinimidyl-6-(biotinamido) hexanoate (HS-LC-Bioin)	SPR	10^6^ CFU/mL	-	-	[135]
	-		-		10^7^ CFU/mL	-	-	[137]
	Chicken carcass wash fluid		Direct reductive amination	Integrated optic interferometer	*Direct assay*: 10^7^ CFU/mL *Sandwich assay*: 10^5^ CFU/mL	10 min	-	[136]
*S*. Typhimurium	Phosphate buffered saline (PBS) Pork	Antibody to CSA-1	Protein G	FRET	10^3^ cells/mL 10^5^ cells/mL	5 min	-	[138]
*S*. Typhimurium *S*. Enteritidis	Poultry	- *Capture*: rabbit polyclonal pAb-anti-*Salmonella* antibody - *Reporter*: rabbit pAb-3238 and mouse anti-*S*. Enteritidis mAb-2F11	Sulfo- N-hydroxysuccinimide (NHS)-LC-Biotin	BARDOT (bacterial rapid detection using optical scattering technology)	10^3^ CFU/mL	12 h	-	[139]
*S*. Typhimurium	Borate buffer & chicken extract	anti-*Salmonella* polyclonal antibodies	Covalent	Quantum dot nanoparticles	10^3^ CFU/mL	30 min	0 to 10^6^ CFU/mL,	[140]
*S*. Typhimurium	phosphate buffer saline	Antibodies against *Salmonella* antigens	Glass/TiO_2_/anti-S-Ab	Titanium dioxide (TiO_2_) nanoparticles Photoluminescence	-	-	10^3^ to 10^5^ cell/mL	[141]
*S*. Enteritidis	Water Milk	DNA	NHS	FRET	10^2^ CFU/mL 1.5 × 10^2^ CFU/mL	2 h	10^2^ to 3 × 10^3^ CFU/mL 1.5 × 10^2^ to 3 × 10^3^ CFU/mL	[142]
	PBS Shredded beef Chicken Turkey breast	- *Capture*: rabbit polyclonal pAb-anti-*Salmonella* antibody - *Reporter*: mouse monoclonal antibodies	Sulfo-NHS-LC-Biotin	Evanescent-based fiber optic sensor	10^3^ CFU/mL 10^7^ to 10^8^ CFU/mL after 18 h of enrichment	2 h	-	[143]
*Salmonella* spp.	Buffer	DNA	Covalent	EIS	-	-	0.1 µM–10 µM	[144]
*Salmonella*	Chicken	Anti-*Salmonella* rabbit pAbs	Dithio-bis-succinimidyl propionate (DSP)	Immunosensors combined with light microscopic imaging system (LMIS)	10^3^ CFU/chicken	-	-	[132]
*S*. Choleraesuis	PBS Whole milk (Test yes/no)	- *Capture*: 5F11-B11 monoclonal antibody - *Detection*: 11D8-D4 monoclonal antibody	*Capture antibody*: deposition onto the LFA *Colloidal gold particles*: sodium citrate chemical reduction	LFA	5 × 10^5^ CFU/mL -	20 h	-	[47]
*S*. Typhimurium *S*. Enteritidis	PBS Chicken (Test of specificity)	- Anti-*Salmonella* rabbit pAbs - Mouse anti–*S*. Typhimurium - Mouse anti–*S*. Enteritidis	Colloidal gold particles Mousse antibodies were applied onto the nitrocellulose membrane	LFA	10^4^ CFU/mL 10^6^ CFU/mL 100% 100%	5–15 min	-	[145]
*S*. Typhimurium	Buffer	Antibody to CSA-1	Carbodiimide	Flow injection amperometry immunofiltration assay	50 cells/mL	35 min	50–200 cells/mL	[46]
*S*. Typhimurium	Chicken carcass washing samples	- Monoclonal fluorescein isothiocyanate labeled anti-*Salmonella* antibody - Polyclonal rabbit anti-*Salmonella* antibody	Biotin	Potentiommetry	119 CFU/mL	15 min	-	[146]
*S*. Typhimurium	Water	Outer membrane porin protein (OmpD)	Carboxilated graphen-graphen oxide	Impedimetry	10 CFU/mL	-	-	[147]
*S*. Enteritidis	Buffer Milk	Biotinylated rabbit anti-*Salmonella* polyclonal antibody	Neutravidin		10^6^ CFU/mL 10^4^ CFU/mL (with nanoparticles) 10^5^ CFU/mL (with nanoparticles)	3 min	-	[148]
*S*. Typhi	Buffer	Rabbit anti-*Salmonella* spp. polyclonal antibody	Covalent		100 CFU/mL	5 min	-	[149]
*S*. Typhimurium	Buffer	Anti-*Salmonella* antibody	Polyethyleneimine	QCM	10^5^ CFU/mL	5 h	10^5^ to 10^9^ CFU/mL	[150]
			Protein A		10^6^ CFU/mL	-	10^6^ to 10^8^ CFU/mL	[151]
			Polyethylenimine-glutaraldehyde and dithiobissuccinimidylpropionate coupling		-	25 min	5.3 × 10^5^ to 1.2 × 10^9^ CFU/mL	[152]
		Polyvalent somatic O antibody of *Salmonella* spp.	Langmuir-Blodgett	AWD	350+/−150 cells/mL	100 s	10^2^ to 10^7^ CFU/mL	[153]
	Chicken breast	Antibody to CSA-1	Protein A	QCM	10^2^ cells/mL (with anti-*Salmonella*-magnetic beads)		ΔF 10^5^–10^8^ cells/mL ΔR 10^6^–10^8^ cells/mL	[154]
	PBS Chicken meat	Mouse monoclonal antibody against *S*. Typhimurium	EDC-NHS		10–20 CFU/mL Validation: good sensitivity	12 min		[155]

QCM: Quartz crystal microbalance; SPR: Surface plasmon resonance; FRET: Fluorescence resonance energy transfer; LFA: Lateral flow assay; AWD: Acoustic wave device.

**Table 3:** Advantages and drawbacks of the biosensors and aptasensors technologies used for the detection of *Salmonella* in food.

	Optical	Lateral Flow Assays	Electrochemical	Mass Based
**Advantages**	- Easy to use - High sensitivity	- Good reproducibility - Very low shelf life - Rapid - Portable - User-friendly - Less interferences - Adequate specificity	- User-friendly - Miniaturization	- High sensitivity - Portable - Rapid - Simple - Stable output
**Drawbacks**	- Pretreatment of sample may be required	- Poor quantitative discrimination - Reproducibility may vary from lot to lot - Low signal intensity - Pretreatment of sample may be required - Mostly qualitative or semi-quantitative	- Low selectivity	- Low sensitivity with liquid samples - Interference induces by nonspecific binding

**Table 4:** Aptamers selected against *Salmonella* strains by the SELEX method.

Microorganism	Aptamers Name	Target for the SELEX	Aptamer Sequences (5′-3′)	Size (Base)	Kd	References
**DNA Aptamers**						
*S*. Typhimurium	33	OMPs	TATGGCGGCGTCACCCGACGGGGACTTGACATTATGACAG	40	-	[172]
	45		GAGGAAAGTCTATAGCAGAGGAGATGTGTGAACCGAGTAA			
	33	OMPs	TATGGCGGCGTCACCCGACGGGGACTTGACATTATGACAG (from Joshi et al. [172])	40	-	[182]
					-	[183]
	S8-7	Whole cell	CTGATGTGTGGGTAGGTGTCGTTGATTTCTTCTGGTGGGG	40	1.73 ± 0.54 μM	[177]
	ST2P	Whole cell	CAAAGATGAGTAGGAAAAGATATGTGCGTCTACCTCTTGACTAAT	87	6.33 × 10^−3^ ± 0.58 × 10^−3^ µM	[178]
	C4	Whole cell	ACGGGCGTGGGGGCAATGCCTGCTTGTAGGCTTCCCCTGTGCGCG	45	-	[179]
*S*. Typhimurium	St1	Whole cell	CCGATGTCCGTTAGGGCTCCTCCATAGAT	29	0.530 ± 0.01 μM	[180]
*S*. Enteritidis	Se-1		CACACCGGAAGGGATGCCACCTAAACCCC	30	4.66 ± 0.35 μM	
	Se-2		CACAGATGACGTCTGGCACATAATTAACAC	30	3.83 ± 0.10 μM	
*S*. Paratyphi A	Apt 22	Whole cell	ATGGACGAATATCGTCTCCCAGTGAATTCAGTCGGACAGCG	41	47 × 10^−3^ ± 3 × 10^−3^ µM	[184]
*S*. Typhimurium	A2	-	CCAAAGGCTACGCGTTAACGTGGTGTTGG	29	-	[185]
*S*. Enteritidis	-	OMPs	TCGGCAACAAGGTCACCCGGAGAAGATCGGTGGTCAAACTGCATAGGTAGTCCAGAAGCCGAACAAGCTGAGGATGAAGAACAACGGCT	89	-	[131]
*S*. Typhi	-	IVB Pili	GGGAACAGUCCGAGCCUCACUGUUAUCCGAUAGCAGCGCGGGAUGAGGGUCAAUGCGUCAUAGGAUCCCGC	71	-	[102]
*S*. Enteritidis	SENT-9	Whole cell	CTCCTCTGACTGTAACCACGCACAAAGGCTCGCGCATGGTGTGTACGTTCTTACAGAGGT	60	7 × 10^−3^ µM	[176]
*S*. Typhimurium	STYP-3	Whole cell	GAGTTAATCAATACAAGGCGGGAACATCCTTGGCGGTGC	39	25 × 10^−3^ µM	[175]
	-	OMPs	TTTGGTCCTTGTCTTATGTCCAGAATGCGAGGAAAGTCTATAGCAGAGGAGATGTGTGAACCGAGTAAATTTCTCCTACTGGGATAGGTGGATTAT (modified from Aptamer 45 of Joshi et al. [172])	96	-	[186,187]
**RNA Aptamers**						
*S*. Typhi	S-PS8.4	IVB pili	UCACUGUUAUCCGAUAGCAGCGCGGGAUGA	30	8.56 × 10^−3^ µM	[173]
*S*. Enteritidis	S 25	Whole cell	GGGUUCACUGCAGACUUGACGAAGCUUGAGAGAUGCCCCCUGAUGTGCAUUCUUGUUGUGUUGCGGCAAUGGAUCCACAUCTACGAAUUC	90	-	[181]

**Table 5:** Aptasensors for *Salmonella* strains detection in food samples.

Microorganism	Sample Matrix	Aptamer Reference	Immobilization Method	Transducer	Limit of Detection	Analyze Time	Working Range	References
*S*. Typhimurium	Buffer	33 from Joshi et al. [172]	Gold surface Thiolated aptamers	SPR	30 CFU/mL	-	10^4^–10^9^ CFU/mL	[189]
		Unknown: obtained from Dr. Srinand Sreevatsan’s group	Gold nanoparticles thiolated aptamers	SERS	10^2^ CFU/mL	45 min	10^2^–10^3^ CFU/mL	[190]
*S*. Paratyphi A	City water	Apt22	Free: DNAzyme	Chemiluminescence	10^4^ CFU/mL	-	10^4^–10^8^ CFU/mL	[184]
*S*. Typhimurium	Buffer	33 from Joshi et al. [172]	Avidin-biotin	Fluorescent	5 CFU/mL	-	10^1^–10^5^ CFU/mL	[191]
		ST2P			25 CFU/mL	-	50–10^6^ CFU/mL	[178]
	Buffer Shrimp samples (Validation)		Free: Flow cytometry		5 × 10^3^ CFU/mL	-	3.8 × 10^4^–3.8 × 10^7^ CFU/mL	[192]
	Buffer Water from Tai Lake (Validation)	33 from Joshi et al. [172]	Streptavidin-biotin	Optical-UV	7 CFU/mL	-	50–10^6^ CFU/mL	[7]
	Buffer	A2	Adsorption		10^5^ CFU/mL	20 min	-	[185]
	Buffer Milk (Validation)	33 from Joshi et al. [172]	Avidin-biotin	Fluorescent	15 CFU/mL	-	10^2^–10^5^ CFU/mL	[193]
*S*. Enteritidis	Milk	-	Streptavidin-biotin	LFA	10^1^ CFU/mL	-	-	[131]
*S*. Typhi	Phosphate buffer	-	EDC-NHS-amine	Potentiometry	-	60 s	0.2–10^6^ CFU/mL	[102]
*S*. Enteritidis	Buffer	SENT-9	Self-assembled monolayer (SAM)	Impedimetry	600 cells/mL	10 min	10^3^–10^5^ CFU/mL	[176]
*S*. Typhimurium		STYP-3				-		[175]
*S*. Typhimurium	Buffer Pork (Validation)	33 from Joshi et al., [172]	Gold nanoparticles thiolated aptamers		3 CFU/mL	-	2.4–2.4 × 10^3^ CFU/mL	[194]
	Buffer	33 from Joshi et al., [172]	Self-assembled monolayer (SAM)		1 CFU/mL	40 min	6.5 × 10^2^ to 6.5 × 10^8^ CFU/mL	[182]
	Eggs						6.5 × 10^3^ to 6.5 × 10^7^ CFU/mL	
	Buffer Apple Juice (Validation)	Aptamer 45 from Joshi et al., [172] with length modification	Covalent		3 CFU/mL	-	10^2^–10^8^ CFU/mL	[186]
			EDC-NHS-amine		6 CFU/mL	-	10^1^–10^8^ CFU/mL	[187]
*S*. Typhimurium	Milk	S8-7 from Dwivedi et al. [177]	Amine	QCM	100 CFU/mL	10 min	100–4 × 10^4^ CFU/mL	[195]
	Buffer Chicken meat	33 from Joshi et al., [172]	Thiolated aptamers – glutaraldehyde - rGO-CHI	DPV	10^1^ CFU/mL	-	10^1^ to 10^6^ CFU/mL	[183]

QCM: Quartz crystal microbalance; SPR: Surface plasmon resonance; SERS: Surface-enhanced Raman spectroscopy; and LFA: Lateral flow assay.
